# Natural resveratrol analogs differentially target endometriotic cells into apoptosis pathways

**DOI:** 10.1038/s41598-023-38692-8

**Published:** 2023-07-15

**Authors:** Agata Gołąbek-Grenda, Mariusz Kaczmarek, Wojciech Juzwa, Anna Olejnik

**Affiliations:** 1 Department of Biotechnology and Food Microbiology, Poznan University of Life Sciences, 48 Wojska Polskiego St., 60-627 Poznan, Poland; 2 Department of Cancer Immunology, Chair of Medical Biotechnology, Poznan University of Medical Sciences, 61-866 Poznan, Poland; 3 Department of Cancer Diagnostics and Immunology, Greater Poland Cancer Centre, 61-866 Poznan, Poland

**Keywords:** Cell biology, Nutrition

## Abstract

The specific characteristics of endometriotic cells are their ability to evade the apoptotic machinery and abnormal response to apoptotic stimuli. Natural-originated compounds may constitute a beneficial strategy in apoptosis modulation in endometriosis. We investigated and compared the potency of natural resveratrol analogs, including piceatannol, polydatin, and pterostilbene, in targeting cell death pathways, including apoptosis-related morphologic and biochemical processes, alongside the modulation of the critical genes expression. Upon resveratrol and pterostilbene treatment, a significant reduction of endometriotic cell viability and an increased apoptotic proportion of cells were noted. The lower antiproliferative potential was found for piceatannol and polydatin. Endometrial stromal T HESC cells were significantly more resistant than endometriotic epithelial 12Z cells to the cytotoxic activity of all analyzed compounds. They differentially affected endometriotic cell viability, cell cycle, anti- and proapoptotic genes regulation, caspases expression and enzymatic activity, and DNA fragmentation. Pterostilbene-mediated endometriotic cell apoptosis modulation was confirmed to be most effective but without evident caspase 3 upregulation. Our study provides valuable insight into the apoptogenic activity of resveratrol and its natural analogs in endometriotic cells. Data obtained revealed the highest therapeutic potential of pterostilbene by effectively targeting cell death determinants in endometriosis, strengthening its optimization in further extensive research.

## Introduction

Endometriosis is an idiopathic condition characterized by ectopic growth and function of endometrial tissue outside the uterine cavity in line with a recent the International Statistical Classification of Diseases and Related Health Problems definition published latterly by WHO^1^. According to Sampson’s concept, menstrual effluents, refluxed into the peritoneal cavity, contain viable endometrial cells that can implant and initiate the formation of endometriotic lesions^2^. The retrograde dissemination of the endometrial cells is a common phenomenon and appears in all women of reproductive age, regardless of whether endometriosis is present^3^. In healthy women, the misplaced endometrial cells are eliminated by the programmed cell death required to maintain cellular homeostasis effectively. The reduced endometrial tissue sensitivity to apoptosis probably causes active ectopic endometriotic lesions development^4^.

Novel therapies focus on various molecular targets, and apoptosis-inducing agents may be a promising therapeutic strategy for endometriosis. Natural plant-derived compounds, including resveratrol is emerging as a promising anticancer agent due to apoptosis induction and cell cycle arrest in various cancer cell types^5,6^. This compound was reported to trigger the molecular proapoptotic cancer-related mechanisms via the mitochondrial-apoptosome-mediated intrinsic pathway and the death receptor-induced extrinsic pathway in different cancers^7^. Anticancer and apoptogenic mechanisms of resveratrol were widely investigated in many in vitro and in vivo studies, which were discussed in several review articles^8,9^. Although the abnormal tissue that grows from endometriosis is not cancerous, it shares several characteristics in terms of the metastasis-like behavior of cells, attachment to other tissues, and invasion of distant body sites^10^. The evidence of shared similar pathogenetic mechanisms of endometriosis and cancers in women may be an avenue for identifying potential targets for future disease management and treatment.

Recently, more attention has been paid to increasing resveratrol bioavailability. The metabolized fraction of resveratrol that reaches the bloodstream and is distributed into the target tissues to exert biological activity is low for its rapid and extensive phase II metabolism by glucuronidation and sulfation on the hydroxyl groups^11^. Scientific interest is emerging in some methoxylated and hydroxylated derivatives of resveratrol to enhance its therapeutic versatility. Due to additional methoxy groups, pterostilbene, a dimethylether analog of resveratrol (3,5-dimethoxy-4′-hydroxystilbene), enhances structural stability and ensures better lipophilicity, absorption, cellular uptake, and bioavailability compared with the maternal compound^12^. Moreover, several studies have focused on the activity of piceatannol (3,3′,4,5′-tetrahydroxystilbene) with higher stability than resveratrol during metabolism^13^. Also, piceatannol has been reported to reduce the high incidence of cardiovascular diseases, exert therapeutic effects in different cancers, and prevent arrhythmia, hypercholesterolemia, angiogenesis, and atherosclerosis^14,15^. Polydatin (3,4′,5-trihydroxystilbene-3-β-D-glucoside), known as piceid, is occupied by a glucopyranoside ring in the C-3 hydroxyl moiety. Glycosylated forms of resveratrol presented greater bioavailability and displayed potent anti-inflammatory and antioxidative activities^16,17^.

In our opinion, extensive studies on the modulation of endometriotic and endometrial cell apoptosis are needed to assess molecular mechanisms underlying the antiproliferative effect and regulation of cell cycle arrest by the natural compound in endometriosis. To our knowledge, we present the first study evaluating the therapeutic potential of natural resveratrol derivatives in endometriosis. We aimed to examine resveratrol, pterostilbene, piceatannol, and polydatin effects on proliferation, cell cycle distribution, and symptomatic apoptosis in the endometriotic 12Z cell line. We also investigated the potential of resveratrol and its derivatives to regulate the gene expression of proapoptotic and antiapoptotic molecules.

## Results

### Effect of resveratrol and its derivatives on endometriotic and endometrial cell viability

**Figure 1:**
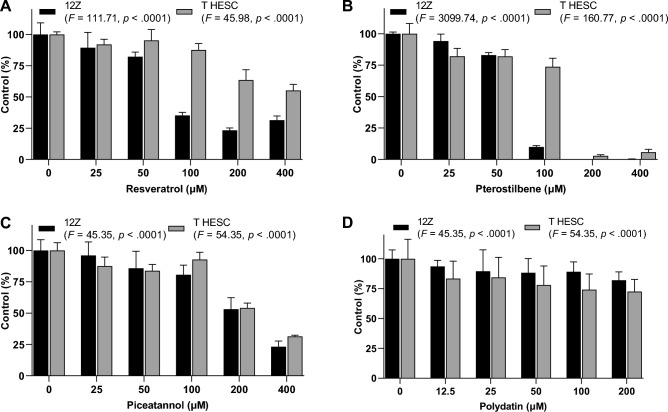
Effect of resveratrol (**A**), pterostilbene (**B**), piceatannol (**C**), and polydatin (**D**) on endometriotic epithelial (12Z) and endometrial stromal (T HESC) cell viability, determined by Alamar Blue reduction assay. The values represent the means (n = 3) ± SD. The significance of the main effects was determined by ANOVA (*p*, *F*).

The effect of resveratrol and its natural analogs on the viability of endometriotic epithelial 12Z cells and endometrial stromal T HESC cells was assessed using the MTT assay*.* Table 1 presents the IC_10_ and IC_50_ values (the inhibitor concentration required for 10% and 50% reduction in enzyme activity) obtained after 48-h incubation with the tested compounds. In 12Z cell culture, the IC_10_ and IC_50_ values of piceatannol and polydatin were significantly higher than those of resveratrol. The cytotoxicity of resveratrol and pterostilbene to 12Z cells was not markedly different. T HESC and 12Z cells varied in susceptibility to the treatment with stilbenes tested, as evidenced by the cytotoxic doses shown in Table 1. Pterostilbene revealed the highest cytotoxic potential for T HESC cells, significantly higher than the parent compound. However, comparing IC_50_ values, pterostilbene cytotoxicity in T HESC cell culture was almost twofold lower than in 12Z cell culture. Similarly, resveratrol evoked much weaker cytotoxic effects in T HESC cells than in 12Z cells. Polydatin at concentrations up to 400 µM was not cytotoxic to the T HESC cells, while at a dose of 86.76 µM, it induced the first cytotoxic symptoms in 12Z cells. It is worth noting the Alamar Blue assay proved a similar cytotoxic effect of analyzed compounds in 12Z and T HESC cells, although it was less sensitive than the MTT assay. The increased susceptibility of endometriotic 12Z cells than endometrial T HESC cells to resveratrol and its natural analogs was consistent in both assays (Fig. 1). To summarize, resveratrol, pterostilbene, and piceatannol had a more apparent toxic effect on endometriotic cells than endometrial cells.

**Table 1:** Effect of resveratrol, pterostilbene, piceatannol, and polydatin on endometriotic epithelial (12Z) and endometrial stromal (T HESC) cell viability, determined by the MTT reduction assay.

Tested compounds	Endometriotic cells—12Z		Endometrial cells—T HESC	
	IC_10_ (µM)	IC_50_ (µM)	IC_10_ (µM)	IC_50_ (µM)
Resveratrol	8.99 ± 4.29	49.32 ± 11.4	160.35 ± 7.05^##^	250.99 ± 1.58^##^
Pterostilbene	29.34 ± 4.46	73.88 ± 6.0	113.79 ± 5.11*, ^##^	145.12 ± 7.71**, ^##^
Piceatannol	167.50 ± 11.36**	223.76 ± 6.47**	222.68 ± 14.49**, ^#^	363.72 ± 16.98**, ^##^
Polydatin	86.76 ± 10.01**	242.85 ± 22.51**	> 400	> 400

*p < 0.05, **p < 0.001 significance of differences between cell treatment with resveratrol analogs compared to resveratrol.

^#^*p* < 0.05, ^##^*p* < 0.001 significance of differences between cytotoxic doses of stilbenes to 12Z and T HESC cells.

### Effects of resveratrol and its analogs on the endometriotic cell monolayer morphology

**Figure 2:**
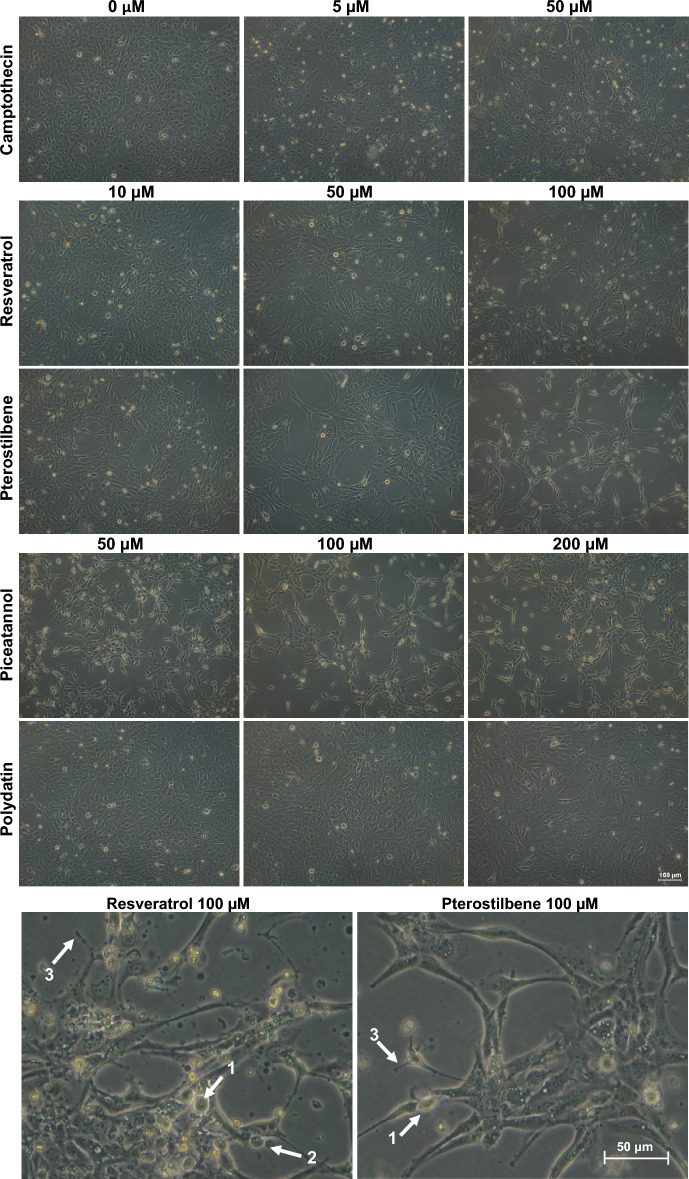
Endometriotic 12Z cell monolayer morphology after 48-h treatment with resveratrol and its analogs. Disturbances in 12Z cell culture developed under treatment with stilbenes were compared with 12Z cell cultures non-treated (negative control) and treated with camptothecin as a reference compound (positive control). Photos were taken at a magnification of 100 × . Arrows indicate cell rounding (1), membrane blebbing (2), and echinoid spikes (3) in endometriotic cell culture under treatment with 100 μM of resveratrol and pterostilbene.

After the treatment of resveratrol and its analogs, morphological changes in the endometriotic cell monolayer were observed under the inverted phase contrast microscope (Fig. 2). As the concentration of the analyzed compound increased, the cell culture became sparsely populated and defective. The most significant disturbances in the development of the endometriotic cell monolayer were detected following treatment with resveratrol and pterostilbene. Inhibition of adherent cell growth, losing contact with adjacent cells, and typical apoptotic features, such as rounding and membrane blebbing, were recorded dependently on the dose of stilbene tested. The arrested proliferation and echinoid spikes on the apoptotic cell surface and rampant death of endometriotic cells were observed, especially after exposition to high resveratrol and pterostilbene dosage. However, the first disturbances in 12Z cell monolayer development were noted after cell treatment with cytotoxic doses as low as 10 µM (Fig. 2).

### Microscopic detection of endometriotic cell apoptosis

**Figure 3:**
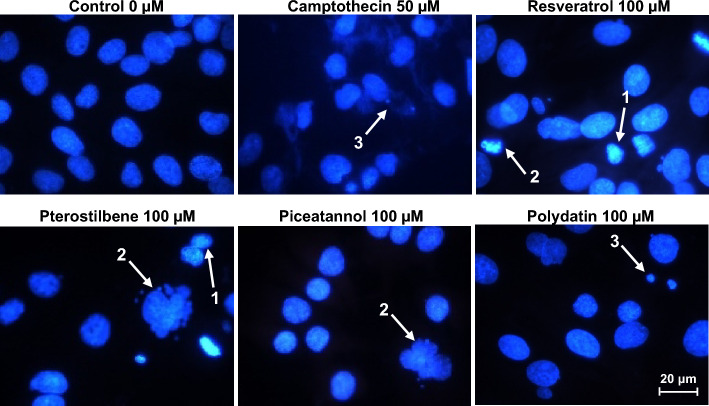
Morphological apoptosis of 12Z cells after treatment with resveratrol and its analogs and staining with DAPI. Control cells have intact nuclei, whereas treated cells depict pyknosis-condensed chromatin (1), nucleus fragmentation (2), and apoptotic bodies (3). White arrows indicate typical features of apoptosis. Photos were taken at a magnification of 400 × .

The apoptogenic properties of the tested compounds were monitored using fluorescence microscopy analysis of treated endometriotic 12Z cells after staining with DAPI (Fig. 3). The cells displayed common characteristics typical for apoptosis, such as chromatin condensation and cleavage, formation of pyknotic bodies of condensed chromatin, and cell disintegration into apoptotic bodies and microvesicles. The results indicated evident changes typical for apoptotic cells, such as increased nuclear condensation, formation of apoptotic bodies, and fragmentation in multiple segregated body features.

### Effect of resveratrol and natural analogs on endometriotic cell death induction

**Figure 4:**
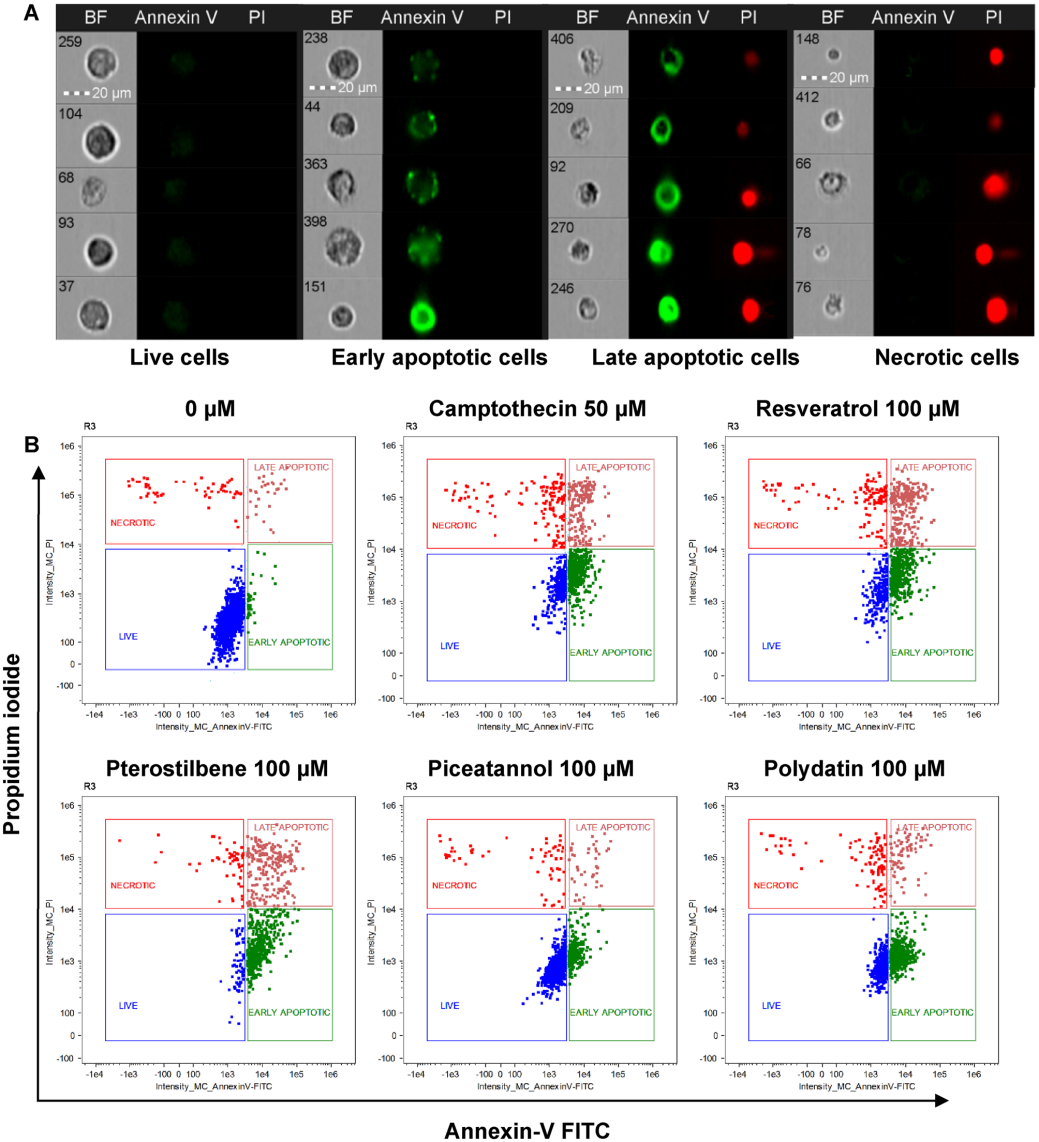

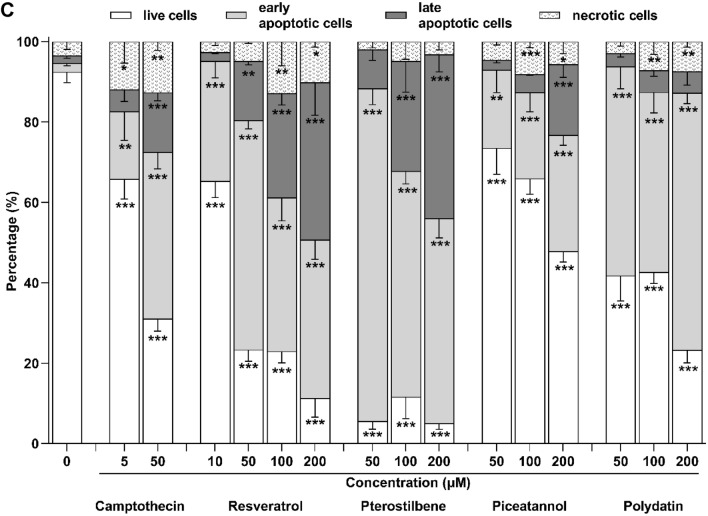
Flow cytometry analysis of endometriotic 12Z cells stained with PI and FITC-Annexin V. Cells were treated with resveratrol, pterostilbene, piceatannol, polydatin and their vehicle (0.5% ethanol) for 48 h. Camptothecin was used as a positive control. (**A**) Representative images of each distinct cellular subset. n = 4; scale bars = 20 μm. (**B**) Representative flow cytometry dot plots. (**C**) Summary of FITC-Annexin V and PI stained populations in 12Z cell cultures. Data show the percentage distribution of four fractions: PI-negative/FITC-Annexin V-negative, PI-positive/FITC-Annexin V-negative, PI-negative/FITC-Annexin V-positive, and PI-positive/FITC-Annexin V-positive. The results are presented as means ± SD. *p < 0.05, **p < 0.01, ***p < 0.001 compared to vehicle control.

After treating endometriotic 12Z cells with stilbenes, the phosphatidylserine externalization was measured by Annexin V and PI double staining (Fig. 4A). Examples of the single-cell images from sub-populations: live cells (Annexin V/PI-double negative), early apoptotic cells (Annexin V-positive/PI-negative), late apoptotic cells (Annexin V/PI-double positive), necrotic cells (Annexin V-negative/PI-positive) are presented in Fig. 4B.

Flow cytometry analysis of the stained cells can distinguish apoptotic cells from necrotic cells. The results in Fig. 4C show that resveratrol and its derivatives dose-dependently decreased the population of live cells and simultaneously increased the population of early and late apoptotic cells with the limited population of mechanical necrotic cells. Under treatment with resveratrol, pterostilbene, and piceatannol at concentrations of 50 µM, 100 µM, and 200 µM, a significant dose-dependent increase in the population of late apoptotic cells was observed in treated 12Z cell cultures. Pterostilbene was the most efficient apoptosis inducer based on the overall apoptotic rate parameter. In the 12Z cell culture exposed to pterostilbene at a dose of 200 µM for 48 h, approximately 94% of treated cells were classified as apoptotic cells. In contrast, 12Z cells treated with polydatin underwent apoptosis with an increased population of early apoptosis cells without induction of late apoptosis (Fig. 4C).

### Effect of resveratrol and natural analogs on DNA fragmentation

**Figure 5:**
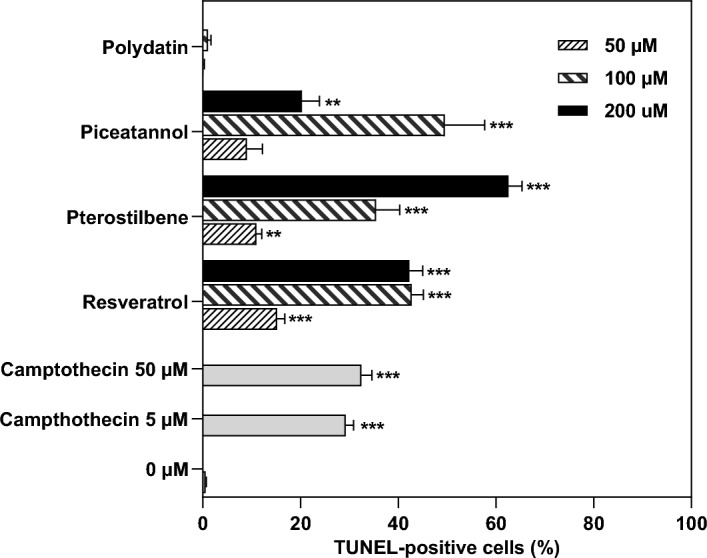
DNA fragmentation in 12Z cells after treatment with resveratrol and its analogs determined using TUNEL flow cytometry analysis. Results of experiments performed in triplicates are presented as means ± SD. *p < 0.05, **p < 0.01, ***p < 0.001 compared to vehicle control.

Cell death via apoptosis in 12Z cells after exposure to resveratrol and its natural derivatives was determined by detecting DNA fragmentation (Fig. 5). DNA degradation was correlated in a dose-dependent manner. TUNEL assay showed increased DNA fragmentation in endometriotic 12Z cells treated for 48 h with resveratrol, pterostilbene, and piceatannol. The most significant DNA strand breaks were observed in the cells exposed to resveratrol and piceatannol at the concentration of 100 µM (42.8% and 49.6%, respectively) and 200 µM pterostilbene (62.6%). In contrast, polydatin did not induce DNA damage in treated 12Z cells (1.08%). Tunel-positive cell population in the 12Z cell culture treated with camptothecin was estimated at 32.5%, compared to 0.6% in the vehicle-treated control culture.

### Effect of resveratrol and its analogs on cell cycle progression

**Figure 6:**
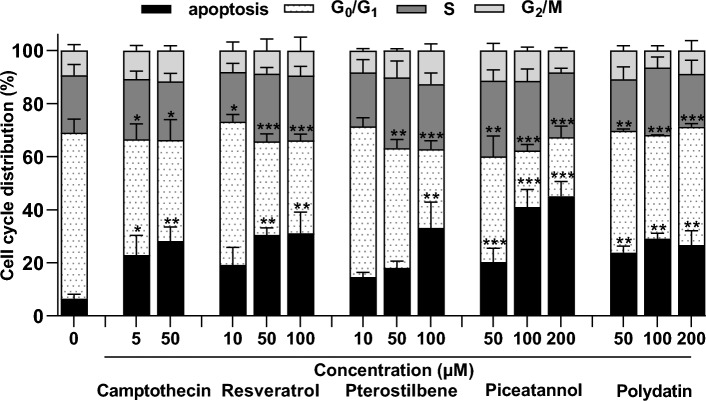
Effect of resveratrol and its analogs on cell cycle progression in endometriotic 12Z cells. Camptothecin was used as a reference apoptosis inducer. The values represent the means (n = 3) ± SD. Significant differences *p < 0.05, **p < 0.01, ***p < 0.001 compared to vehicle control.

To investigate whether resveratrol and its analogs have interfered with cell cycle progression in endometriotic 12Z cells, we analyzed the cell cycle distribution by flow cytometry. The obtained data showed that treatment with resveratrol and its analogs caused a significant increase in an apoptotic cell population with lower DNA content and simultaneously a decrease in cell population in G_1_/G_0_ cycle phase depending on the compound concentration, except for polydatin, which did not increase the number of apoptotic cells in a dose-dependent manner (Fig. 6). The largest subpopulation of apoptotic cells was found in the cell cultures treated with resveratrol and pterostilbene at the concentration of 100 µM (31.2 ± 8.0% and 33.1 ± 9.7%, respectively) and piceatannol at a concentration of 200 µM (45.1 ± 5. 6%).

### Effect of resveratrol and its analogs on the expression of genes involved in apoptosis

**Figure 7:**
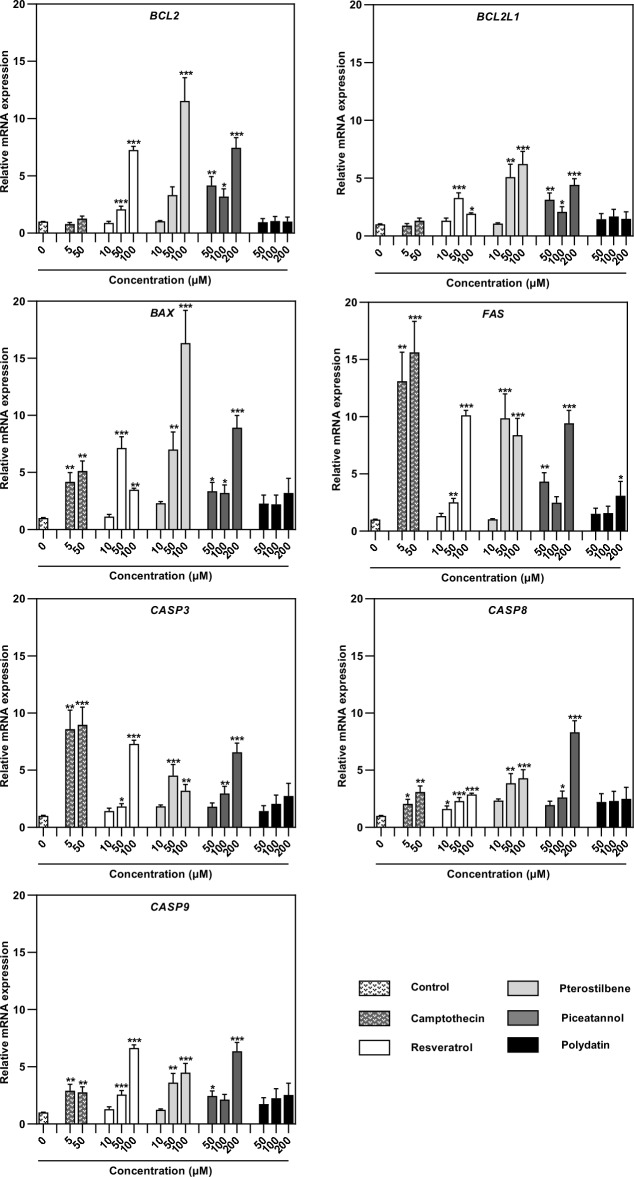
Effect of resveratrol and its analogs on the mRNA expression of *BCL2*, *BCL2L1*, *BAX*, *FAS*, *CASP3*, *CASP8*, and *CASP9* genes in endometriotic 12Z cells, assessed by real-time PCR. Data were normalized against *β-actin* and expressed as relative mRNA expression (-fold of control). The values represent the means (n = 3) ± SD. Significant differences *p < 0.05; **p < 0.01; ***p < 0.001 compared to vehicle control.

The effect of resveratrol and its natural analogs on the expression of pro- or antiapoptotic regulators, was examined by real-time PCR. The analysis of mRNA expression showed that the analyzed compounds affect the expression of several proapoptotic genes and genes of two pro-survival proteins. The enhanced expression of pro-survival B-cell lymphoma 2 (*BCL-2*) and BCL2-like 1 (*BCL2L1*) genes was observed in 12Z cells treated with resveratrol (50 µM and 100 µM), pterostilbene (50 µM and 100 µM), and piceatannol (50 µM, 100 µM, and 200 µM) (Fig. 7). On the other hand, these stilbenes significantly increased the mRNA expression of the *BAX* gene, which promotes apoptosis. The highest *BAX* mRNA expression was observed in 12Z cells treated with pterostilbene at a concentration of 100 µM; in this treatment, the *BAX* expression level increased approximately 16-fold. *BAX* overexpression was also documented in 12Z cells exposed to resveratrol. This compound at a dose of 50 µM revealed a similar capacity to enhance *BAX* mRNA expression, increasing *BAX* transcripts by approximately sevenfold. The results indicate that the analyzed compounds can initiate cell death by varying the balance between *BAX* and *BCL-2*.

Moreover, after 12Z cell treatments with resveratrol and pterostilbene (50 µM and 100 µM) and piceatannol (200 µM), the mRNA expression of death receptor FAS was significantly increased. The *FAS* overexpression depended on the concentration of stilbenes, reaching a relative expression up to tenfold higher in treated endometriotic cells. The role of proteases in resveratrol-, pterostilbene-, and piceatannol-induced apoptosis was also indicated by the upregulation of well-known initiator caspases of the mitochondrial and death receptor apoptotic pathways such as caspases 8, 9 and executioner caspase 3 (Fig. 7). Polydatin did not significantly increase mRNA transcripts of the analyzed *BAX*, *FAS*, *CASP3*, *CASP8*, and *CASP9* genes.

### Effect of resveratrol and its analogs on the activity of caspases-3/7

**Figure 8:**
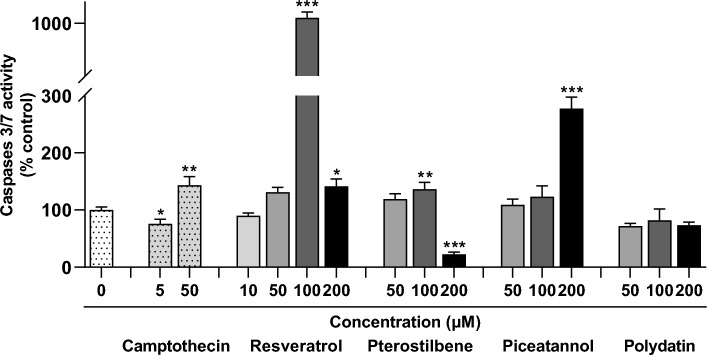
Effect of resveratrol and its analogs on caspases-3/7 activation. The values represent the means (n = 3) ± SD. Significant differences *p < 0.05, **p < 0.01, ***p < 0.001 compared to vehicle control.

The effect of resveratrol and its analogs on the effector caspases-3/7 activity in endometriotic cells is shown in Fig. 8. Resveratrol evoked a dose-dependent caspase-3/7 activation in 12Z cells (F = 2467.29, *p* < 0.0001). The most potent up-regulating effect was observed under treatment with resveratrol at a concentration of 100 µM, which caused a 10.3-fold increase in caspase-3/7 activity. It was consistent with the results of *CASP3* gene expression activation. As shown in Fig. 8, pterostilbene at a concentration of 100 μM modestly activated caspases 3/7, although it inhibited their activity at 200 μM. There was a 2.8-fold activation of caspase 3/7 enzymes following treatment with piceatannol at a 200 μM dose, which complies with the *CASP3* mRNA expression level. Caspase 3/7 activity was not significantly changed in the 12Z cells by their exposure to polydatin.

## Discussion

An important clue for developing new therapeutic strategies in endometriosis is that the eutopic and ectopic endometrium of endometriotic patients shows less susceptibility to apoptosis than the endometrium of non-endometriotic patients^18^. For this reason, manipulating programmed cell death processes by enhancing apoptotic stimulants of endometriotic cells could be a promising approach to treating endometriosis. This study was designed to evaluate the effects of natural analogs of resveratrol on the inhibition of endometriotic cell survival pathways and upregulation of cell death pathways, including apoptosis signaling.

The present study determined the cytotoxic effect of the analyzed stilbenes on human endometrial stromal cells (T HESC) and human endometriotic epithelial cells (12Z). The 12Z cell line retains characteristics of the active phase of endometriosis and is considered suitable for studying the cellular and molecular context of endometriosis^19^. In contrast, the T HESC line represents a distinct stromal compartment of the endometrium and resembles typical endometrial features; therefore, it has been widely applied in endometriosis studies^20,21^. However, endometriotic stromal cells were not employed in the experiments due to the lack of an established immortalized stromal cell line. Recently, the 22B cell line derived from the stromal compartment of endometriotic tissue was no longer available for current research because of the cessation of cell growth during culture^22^. A more recent study by Madanes et al. described the effect of resveratrol on human endometrial stromal St-T1b and epithelial 12Z cells^23^. Resveratrol at a concentration of 100 µM after 48-h treatment was found to reduce cell viability by 45.4% and 49.2% in 12Z and St-T1b cell cultures, respectively. The effects of resveratrol were analyzed on primary cultures of human endometrial epithelial cells obtained from eutopic endometrial biopsies of patients with endometriosis. After exposure to 50 µM and 100 µM resveratrol, cells from control women and endometriosis patients showed a significantly lower proliferative potential^24^. In other studies, resveratrol at concentrations up to 100 μM did not influence the viability of endometrial stromal cells derived from patients with endometriosis after 48-h treatment. Increasing the dose of resveratrol to 200 μM reduced endometriotic stromal cell viability to approximately 50%^25,26^. Resveratrol by itself did not induce apoptosis in the ectopic stromal cells; however, it enhanced the effect of TNF-α-related-apoptosis-inducing ligand (TRAIL) and acted by silencing survivin expression^26^. The control non-endometriotic T HESC cells derived from human endometrium were also applied in our cytotoxicity experiments since the distinction in the cellular response between endometrial and endometriotic tissue is an important issue in evaluating the activity of the therapeutic agent. Endometrial T HESC cells were more resistant to their cytotoxic activity than endometriotic 12Z cells. Based on the IC_50_ values, the cytotoxicity of resveratrol was fivefold higher for the endometriotic 12Z cells than for the endometrial T HESC cells. Resveratrol treatment seems to have a different effect on endometrial and endometriotic cells, with a harmful impact directed to endometriotic cells. Nonetheless, diverse cell population compartment representativeness must be accentuated in assessing the susceptibility of endometriotic and endometrial cells.

The results presented in this work indicated that natural resveratrol analogs are cytotoxic to cells derived from the endometrium and endometriotic lesions with different degrees of potency. Pterostilbene, a dimethyl ether analog of resveratrol with better intestinal absorption and elevated hepatic stability, has been reported to regulate pathogenic pathways associated with carcinogenesis, hematologic diseases, neurological, and age-related disorders^27^. Pterostilbene inhibited the growth of highly malignant melanoma cells at a bioavailable concentration by interfering with the molecular signaling during cell division. Moreover, pterostilbene demonstrated activity in lymphoma cell lines with a mutation of the Fas gene, resistant to apoptosis induced by resveratrol and piceatannol^28^. Compared to resveratrol, fewer hydroxyl groups result in less susceptibility to conjugation metabolism; therefore, a longer half-life is predicted for pterostilbene^29^. However, until now, pterostilbene has not been investigated as an anti-endometriotic agent. Its cytotoxic effect on endometriotic 12Z cells was lower than resveratrol. On the other hand, pterostilbene inhibited the proliferation of endometrial T HESC cells more potently than the parent compound.

Plant-derived piceatannol has been identified as an antileukemic compound and a potent inhibitor of the proliferation and migration of vascular endothelial cells. As a result, piceatannol can limit the development of tumors by inhibiting neovascularization. Polydatin, the glycosylated form of resveratrol, can increase the quantity of trans-resveratrol due to the hydrolysis by β-glucosidase secreted by bacteria in the colon. In recent years, several studies have suggested that polydatin possesses a higher capacity than resveratrol to alleviate oxidative stress and has a more potent anti-inflammatory effect^30^. It was hypothesized that the additional hydroxyl group enhances the piceatannol reactivity and makes it a more potent free radical scavenger than resveratrol^28^. However, interference in various cellular targets and proven antitumorigenic effects^14,31^, piceatannol and polydatin biological activity are not yet determined in endometriosis. The potentially bioactive nature of the resveratrol analogs strongly justifies the research undertaking on their preventive and therapeutic effects on endometriosis. However, comprehensive and extensive research is needed to clarify molecular and cellular regulations of endometriotic cell growth and expansion treated with the proposed new alternative compounds.

We observed that under treatment with resveratrol and its analogs, the percentage of positive cells for Annexin V, an early sign of apoptosis, was increased significantly. Still, the most efficient increase in the population of apoptotic cells was caused by pterostilbene. Cumulative evidence has pointed out that pterostilbene shows stronger antiproliferative and apoptotic effects than resveratrol in human cervical cancer cells^32,33^. The experiments with pterostilbene ascertain it acts as a robust agent capable of regulating the p53-dependent apoptotic pathway and targeting the Nrf-2 pathway by developing endoplasmic reticulum-mediated stress in cervical cancer cells^32,34^. The superior inhibitory effects of pterostilbene compared to resveratrol were associated with the induction of p53 and p21, which contribute to cell cycle arrest at S and G_2_/M phases and subsequent reduction of cyclin E1 and cyclin B1^33^.

Our findings indicate that the first signs of an increase in the incidence of apoptosis were observed at 50 µM concentration of all analyzed compounds, identified by oligonucleosomal DNA fragmentation and accumulation on the histogram as subG1 apoptosis fraction. The DNA strand breaks represent a hallmark of the later stages of apoptosis, which are mediated by several endonucleases. Caspase-activated DNase (CAD) is expressed as a heterodimer with its inhibitor ICAD, cleaved by caspase-3 leading to CAD activation, degrades the nuclear material^35^.

We analyzed the activation of caspases at the molecular and cellular levels. Resveratrol upregulated caspase-3 mRNA expression and proteolytic activity by 7.3- and 10.3-fold, respectively. Caspase 3 expression in pterostilbene-induced apoptosis in endometriotic cells was much weaker, even though this compound generated the most significant number of DNA strand breaks. Data on the apoptotic activity of resveratrol and its natural analogs for human T lymphoma cells revealed that the pan-caspase-inhibitor Z-VAD-fmk could inhibit apoptosis induced by trans-resveratrol, piceatannol, and 3′-hydroxypterostilbene but not apoptosis induced by pterostilbene, suggesting that it could activate apoptosis through a caspase-independent mechanism^36^. It has now been recognized that in response to apoptotic stimuli, mitochondria can also release caspase-independent cell death effectors such as apoptosis-inducing factor (AIF) and endonuclease G. Following release and translocation to the nucleus, AIF becomes an active executioner of the cell death by the initiation of large-scale DNA fragmentation^37^. It is essential to realize that apoptosis evolved complex death pathways, which overlap with each other. The traditional pathway involving BCL family proteins and caspases may recruit an additional response mechanism, such as caspase-independent AIF release, to facilitate the completion of apoptosis^37^. Overexpression of BAX was shown to trigger the release of AIF in 293 T and HeLa cells, suggesting that it can participate in multiple death paradigms^38^. According to this statement, in our study, pterostilbene was found to enhance *BAX* expression significantly. Different proapoptotic potentials between resveratrol and pterostilbene presented in this work are probably due to the structure–activity relationships. It has been found that the 3,5-dimethoxy motif facilitated the apoptogenic activity of the compound, even in cells expressing the multidrug-resistant phenotype or the antiapoptotic oncogene^36^.

An interesting inhibitory effect of piceatannol on the endometriotic cells was observed in our study. The experimental data suggest that apoptosis induction by piceatannol could be related to the extensive fragmentation of nuclear DNA in the treated 12Z cells, despite a relatively high IC_50_ value (223.76 µM) indicating lower cytotoxic potency than resveratrol and pterostilbene. The only difference between the resveratrol and piceatannol is the presence of an additional hydroxy group in one of the piceatannol's aromatic rings. Piceatannol was found to exhibit potent tyrosine kinase inhibitory activity and inhibit a variety of tyrosine kinases involved in cell proliferation and overexpressed in different cancer cells^39^. Data obtained show that piceatannol at 100 µM led to almost 50% internucleosomal DNA fragmentation in 12Z cells with a significant increase in caspase-8, -9, and -3 mRNA expression. Similar observations concerning a minor piceatannol cytotoxic effect were made in hepatoma HepG2 cells at piceatannol doses ranging from 0 to 200 µM after 24, 48, and 72 h of treatment. Likewise, a significant percentage of apoptotic cells was detected in HepG2 cells treated with piceatannol at 100 µM and 200 µM, which also confirmed the TUNEL assay^40^. Our findings indicate that resveratrol, pterostilbene, and piceatannol at higher concentrations up-regulated the expression of the antiapoptotic *BCL2* gene, and pterostilbene evoked the most substantial effect. Protein BCL-2 resides in the outer mitochondrial wall and inhibits cytochrome *c* release^41^. Pterostilbene was previously found to increase the expression of antiapoptotic BCL-2 and activate MAPK/ERK and PI3K/AKT signal pathways in human neuronal SH-SY5Y cells in neuroprotective experiments. Pterostilbene, which exhibits estrogenic activity, mediated neuroprotective potential via mainly ER-α receptor, which activated the BCL-2 expression presumably through genomic estrogen signaling^42^. The distribution of hydroxyl groups in aromatic rings in the stilbenes makes them similar in chemical structure to endogenous and synthetic estrogens such as 17β-estradiol (E2) or diethylstilbestrol^43^. One early identified biological target of resveratrol was the estrogen receptor (ER), and the compound was classified as a phytoestrogen. After dimerization of ER receptors and translocation to the nucleus, they bind to estrogen response elements (ERE) within target genes. The transcription of genes by ER can also be mediated through the non-classical genomic pathway by its direct binding to DNA with different transcription factors (e.g., AP-1, SP-1, or NF-κB) without binding to ERE. Genes involved in proliferation and survival or apoptosis, such as cyclin D1 and *BCL2*, are regulated by ER binding to the SP-1^44^. Many estrogenic compounds display varying degrees of agonism/antagonism depending on the cell type and target gene, and they are referred to as selective estrogen receptor modulators (SERMs). Resveratrol was thought to affect breast cancer cells through ER-dependent signaling pathways, some studies demonstrated that resveratrol analogs decreased triple‑negative breast cancer cell viability in a dose-dependent manner, indicating the existence of an alternative, ER-independent mechanism^45^. The well-characterized endometriotic epithelial 12Z cells used in our experiments are ER-positive (ER-α, ER-β)^46^. Endometrial stromal T HESC cells were also found to express ER and were applied in analyzing the regulation of steroid receptors upon treatment with their antagonists and induction of decidualization^47,48^. Affecting mediators down-regulating ER signaling constitutes an essential direction for therapeutic purposes because estradiol at strikingly high quantities is the master regulator of endometriotic tissue pathology^49^.

Polydatin represents a natural precursor of resveratrol, in which the hydroxyl is replaced and occupied by a glucopyranoside ring, enabling much better bioavailability than resveratrol, benefiting from the way entering cells via glucose carriers^50^. A number of studies have suggested that polydatin therapeutic effect originates from its anti-inflammatory^51^, antioxidant^52^, and anticancer^53^ activities. It remains unclear whether polydatin, like resveratrol, has antioxidation and antitumor properties or constitutes a clinically advantageous approach. In our studies, the effect of polydatin on endometriotic cell apoptosis was relatively minor. All analyzed compounds showed higher inhibition capacity in cell viability than polydatin did. Even though polydatin induced the early apoptosis stage under all measured concentrations (50 µM, 100 µM, 200 µM), cells did not go into the late apoptotic phase as under treatment with other stilbenes. Dose-independently, polydatin unaffected neither caspase activation nor expression of proapoptotic proteins. The reason that polydatin shows lower biological activity than resveratrol at the same concentration was investigated in the different tumor cells. The results indicated that polydatin was harder uptaken by the cells than resveratrol, and probably specific transporters in cells or lipid rafts endocytosis may determine its bioactivity^54^.

Active compounds from natural products hold, in general, multiple molecular targets and can affect different signaling pathways involved in proliferation, apoptosis, invasion, migration, inflammation, reactive oxidative stress, and angiogenesis in the complex disease such as endometriosis^6^. New developed therapeutics for endometriosis will be based on the prevention of off‐target toxicities like side effects on the eutopic endometrium or function of the reproductive tract. Therefore, the long‐term safety of natural products, target pathway selectivity, and prevent recurrence will be assessed in future studies. Moreover, challenges should be addressed to improve bioavailability by implementing prodrugs, solid dispersions, and lipid‐based formulation technologies^55^. Recently, some locally administered platforms were proposed to deliver drugs for endometriosis treatment, utilizing delivery technologies already explored in cancers. A polymeric drug delivery system to control the prolonged release of curcumin from nanofibers in the peritoneum and pelvic cavity was developed in a mouse model of endometriosis. Besides, in the rat endometriosis model, polymer/nucleic acid conjugates reduced the ectopic endometrial lesions in ectopic locations. The small-molecule drugs or siRNA can be targeted to the ectopic endometriotic tissue by the micelle systems and polymeric or peptide conjugates. Different nanoparticle structures (metal-oxide nanoparticles, silver nanoparticles) can be designed to recognize specific receptors in the lesions' microenvironment and target drug delivery^56^. Endometriosis drug delivery technologies are a relatively new approach but are essential for clinically translatable therapeutics for women's health applications.

As discussed above, the study results indicate that resveratrol and its natural analogs may offer substantial preventive and therapeutic potential for treating endometriosis by specific induction of apoptosis via death-signaling pathways. It is worth emphasizing that non-endometriotic T HESC cells were significantly less affected by resveratrol and its key phytoestrogenic analogs. The significant effects of pterostilbene on the proapoptotic events contributed to considerable inhibition of endometriotic cell proliferation, which was more preferential than resveratrol. The anti-endometriotic potency of pterostilbene has been unveiled and described for the first time in the presented work. Given the previous reports and our insights, we assumed more attention should be paid to the estrogenic activity caused by natural phytoestrogens in evaluating their bioactivity. We conclude that the properties of resveratrol and pterostilbene, involving the selective suppression of endometriotic cell proliferation and induction of apoptosis, support their preventive and therapeutic activity against endometriosis.

## Materials and methods

Unless specified otherwise, all chemicals were purchased from Merck KGaA (Darmstadt, Germany).

### Endometriotic and endometrial cell cultures and treatment

The immortalized human endometriotic epithelial cells (12Z), derived from peritoneal endometriosis lesions, were obtained from Applied Biological Materials Inc. (Richmond, Canada). The 12Z cells were cultured in Dulbecco's Modified Eagle Medium/Nutrient Mixture F-12 (DMEM/F12), containing 10% fetal bovine serum (FBS) and 50 mg/L gentamycin. American Type Culture Collection (ATCC, Manassas, VA, USA) was the source for human endometrial stromal cells immortalized by telomerase (T HESC, CRL-4003). The cells were maintained routinely in phenol red-free DMEM/F12 supplemented with 10% charcoal/dextran treated FBS, 1% ITS + 1, and 50 mg/L gentamycin, at 37 °C and 5% CO_2_. Both cell lines were negative for mycoplasma contamination; mycoplasma-free cells were confirmed repeatedly during the study.

Cells were seeded at a density of 0.15 × 10^5^ cells/cm^2^ in 6-well plates. The 24-h cell cultures were treated with resveratrol and its derivatives for 48 h under standard culture conditions. Camptothecin was applied as a reference apoptosis inducer; its final concentrations were set to 5 µM and 50 µM.

### Cell viability assay

Cell viability and metabolic activity were determined using the MTT colorimetric assay, according to the protocol described in previous studies^57^. Based on the obtained data, the dose–response curves were plotted, and inhibitory concentrations (IC) were calculated. In addition, cell proliferation was measured using the alamarBlue™ Cell Viability Reagent (Invitrogen, Carlsbad, CA, USA) according to the manufacturer's protocol. Fluorescence was measured using a Tecan M200 Infinite microplate reader (Tecan Group Ltd., Männedorf, Switzerland).

### Microscopic detection of cell apoptosis

After treatment, cell monolayer morphology was assessed under an inverted phase contrast microscope Axiovert 40C (Zeiss, Germany). Moreover, 12Z cells cultured on Millicell EZ SLIDE glass (Millipore, Merck KGaA) were subjected to the standard treatment protocol. Following exposure, cells were fixed with 4% paraformaldehyde, washed with PBS, permeabilized with 0.1% Triton X-100, and stained with 1 μg/ml DAPI. The cell nuclear morphology was assessed by fluorescence microscopic inspection (Axiovert 200, Zeiss, Germany).

### Annexin-V/PI staining assay

Apoptosis was determined by Annexin V and propidium iodide (PI) double staining with the FITC Annexin V Apoptosis Detection kit (BD Pharmingen, Heidelberg, Germany) by flow cytometry according to the manufacturer's instructions. Briefly, cells were harvested and washed with PBS; then, they were suspended in the binding buffer to obtain 1 × 10^6^ cells/ml concentration and stained with FITC Annexin V and PI. The cells were analyzed with an Amnis™ FlowSight™ flow cytometer (Luminex Corporation, TX, USA). Cell populations were categorized into four subpopulations: live cells (Annexin V/PI-double negative), early apoptotic cells (Annexin V-positive/PI-negative), late apoptotic cells (Annexin V/PI-double positive), necrotic cells (Annexin V-negative/PI-positive).

### TUNEL assay

The detection of DNA fragmentation in 12Z cells was performed using the terminal deoxynucleotidyl-transferase-mediated dUTP nick end labeling (TUNEL) assay with In Situ Cell Death Detection Kit (Roche Diagnostics, Indianapolis, IN, USA). The test detects DNA breakage that is identified by terminal deoxynucleotidyl transferase. After treatment, the harvested cells were fixed in 4% paraformaldehyde and permeabilized in 0.1% Triton X-100. After washing in PBS, cells were subjected to TUNEL labeling with fluorescein-labeled dUTP and terminal deoxynucleotidyl transferase mixture. Cells were analyzed by flow cytometry using an Amnis™ FlowSight™ flow cytometer.

### Cell cycle analysis

Cell cycle progression was analyzed by flow cytometry using PI staining. After treatment, 12Z cells were collected, washed with PBS, and fixed in 70% ethanol. The cells were then washed, resuspended in PBS, and stained with 50 μg/ml PI in the presence of 100 μg/ml RNase A. Cell cycle distribution was analyzed using a FACSCanto flow cytometer (Becton Dickinson, San Jose, CA, USA). Data analysis was performed with the FACS Diva software (Becton Dickinson).

### Caspase-3/7 activity

To quantitate the proteolytic activity of caspase 3 and 7, we performed an assay based on the proteolytic cleavage of the C-terminal side of the aspartate residue in the DEVD peptide substrate by caspase 3/7 and into the fluorescent rhodamine 110. Caspase 3/7 activity was determined using the APO-One Homogenous Caspase-3/7 Assay (Promega Corporation, Wisconsin, USA) following the manufacturer's instructions. The obtained data were normalized to cellular protein content. The total protein was quantified by BCA assay (Pierce® BCA Protein Assay Kit, Thermo Scientific Inc., USA) according to the manufacturer's protocol.

### RNA extraction and real-time PCR

Gene expression analysis was carried out according to the previously described protocol^58^. TRI reagent was applied for total RNA isolation, synthesis cDNA Transcriptor First-Strand kit (Roche Diagnostics GmbH, Mannheim, Germany) for first-strand cDNA synthesis, and SYBR® Select Master Mix (Life Technologies, Carlsbad, CA, USA) for real-time PCR. The primers used for the amplification of cDNAs are listed in Table 2. The levels of transcripts were normalized using β-actin as an internal control, which was previously applied in 12Z cells^59^. Relative mRNA expression was shown as fold change calculated using the 2^−ΔΔCt^ method compared with control cells.

**Table 2:** The primers sequence used for real-time PCR.

Gene	Accession	No. Sequence (5′–3′)	Amplicon (bp)
Hs *BCL2*	NM-000633.3	F: ATC GCC CTG TGG ATG ACT GAG T R: GCC AGG AGA AAT CAA ACA GAG GC	127
Hs *BCL2L1*	NM-138578.3	F: GCC ACT TAC CTG AAT GAC CAC C R: AAC CAG CGG TTG AAG CGT TCC T	131
Hs *BAX*	NM-001291428.2	F: TCA GGA TGC GTC CAC CAA GAA G R: TGT GTC CAC GGC GGC AAT CAT C	103
Hs *FAS*	NM-152872.4	F: GGA CCCA GAA TAC CAA GTG CAG R: GTT GCT GGT GAG TGT GCA TTC C	125
Hs *CASP3*	NM-004346.4	F: GGA AGC GAA TCA ATG GAC TCT GG R: GCA TCG ACA TCT GTA CCA GAC C	146
Hs *CASP8*	NM-001372051.1	F: AGA AGA GGG TCA TCC TGG GAG A R: TCA GGA CTT CCT TCA AGG CTG C	142
Hs *CASP9*	NM-001278054.2	F: GTT TGA GGA CCT TCG ACC AGC T R: CAA CGT ACC AGG AGC CAC TCT T	129

### Statistical analysis

All data are presented as means ± SD from three independent replications. Statistical analysis was performed using STATISTICA version 13.3 software (Statsoft, Inc., Tulsa, OK, USA). The Shapiro–Wilk test was used to assess distributional assumptions, and the equality of variances hypothesis was verified with Levene’s test. A Student’s *t*-test was used to compare two groups of data. One-way analysis of variance (ANOVA) followed by Tukey’s post hoc test was performed to determine the differences between the mean values of multiple groups. *p* ≤ 0.05 was the cut-off point for a significant difference.

## Data Availability

The datasets generated during and/or analyzed during the current study are available from the corresponding author on reasonable request.

## Abbreviations

AIF: Apoptosis-inducing factor
BAX: BCL2 associated X protein
BCL-2: B-cell lymphoma 2
BCL2L1: BCL-2-like protein 1
BID: BH3-interacting domain death agonist
BOK: BCL-2-related ovarian killer protein
CAD: Caspase-activated DNase
CASP: Caspase
CDKs: Cyclin-dependent kinases
CYB1B1: Cytochrome P450 family 1 subfamily B member 1
ER: Estrogen receptor
ERE: Estrogen response elements
E2: 17β-Estradiol
FAS: Tumor necrosis factor receptor superfamily member 6
FASL: FAS ligand
MAPK/ERK: Mitogen activated protein kinase extracellular-signal-regulated kinase
PI3K/AKT: Phosphatidylinositol 3-kinase/protein kinase
Nrf-2: Nuclear erythroid 2-related factor 2
TNF-α: Tumor necrosis factor α
TRAIL: TNF-related apoptosis-inducing ligand

## References

[1] ICD-11. International Classification of Diseases and Related Health Problems (ICD-11). International Classification of Diseases and Related Health Problems tool (Version 11); 2020. https://icd.who.int. Accessed 14 January 2023. (2020).

[2] Sampson J (1924). Benign and malignant endometrial implants in peritoneal cavity, and their relation to certain ovarian tumors. Surg. Gynecol. Obstet..

[3] Halme J, Hammond MG, Hulka JF, Raj SG, Talbert LM (1984). Retrograde menstruation in healthy women and in patients with endometriosis. Obstet. Gynecol..

[4] Nasu K (2011). Aberrant expression of apoptosis-related molecules in endometriosis: A possible mechanism underlying the pathogenesis of endometriosis. Reprod. Sci..

[5] Heo J-R, Kim S-M, Hwang K-A, Kang J-H, Choi K-C (2018). Resveratrol induced reactive oxygen species and endoplasmic reticulum stress-mediated apoptosis, and cell cycle arrest in the A375SM malignant melanoma cell line. Int. J. Mol. Med..

[6] Gołąbek A, Kowalska K, Olejnik A (2021). Polyphenols as a diet therapy concept for endometriosis-current opinion and future perspectives. Nutrients.

[7] Ko J-H (2017). The role of resveratrol in cancer therapy. Int. J. Mol. Sci..

[8] Rauf A (2018). Resveratrol as an anti-cancer agent: A review. Crit. Rev. Food Sci. Nutr..

[9] Zhang L-X (2021). Resveratrol (RV): A pharmacological review and call for further research. Biomed. Pharmacother..

[10] Kapoor R, Stratopoulou CA, Dolmans M-M (2021). Pathogenesis of endometriosis: New insights into prospective therapies. Int. J. Mol. Sci..

[11] Walle T, Hsieh F, DeLegge MH, Oatis JE, Walle UK (2004). High absorption but very low bioavailability of oral resveratrol in humans. Drug Metab. Dispos..

[12] Nawaz W (2017). Therapeutic versatility of resveratrol derivatives. Nutrients.

[13] Setoguchi Y (2014). Absorption and metabolism of piceatannol in rats. J. Agric. Food Chem..

[14] Seyed MA, Jantan I, Bukhari SNA, Vijayaraghavan K (2016). A Comprehensive review on the chemotherapeutic potential of piceatannol for cancer treatment, with mechanistic insights. J. Agric. Food Chem..

[15] Banik K (2020). Piceatannol: A natural stilbene for the prevention and treatment of cancer. Pharmacol. Res..

[16] Ravagnan G (2013). Polydatin, a natural precursor of resveratrol, induces β-defensin production and reduces inflammatory response. Inflammation.

[17] Potdar S, Parmar MS, Ray SD, Cavanaugh JE (2018). Protective effects of the resveratrol analog piceid in dopaminergic SH-SY5Y cells. Arch. Toxicol..

[18] Delbandi A-A (2020). Evaluation of apoptosis and angiogenesis in ectopic and eutopic stromal cells of patients with endometriosis compared to non-endometriotic controls. BMC Women’s Health.

[19] Gołąbek-Grenda A, Olejnik A (2022). In vitro modeling of endometriosis and endometriotic microenvironment—challenges and recent advances. Cell. Sig..

[20] Sui C (2016). PAI-1 secretion of endometrial and endometriotic cells is Smad2/3- and ERK1/2-dependent and influences cell adhesion. Am. J. Transl. Res..

[21] Horné F (2019). Impaired localization of claudin-11 in endometriotic epithelial cells compared to endometrial cells. Reprod. Sci..

[22] Samartzis EP, Fink D, Stucki M, Imesch P (2019). Doxycycline reduces MMP-2 activity and inhibits invasion of 12Z epithelial endometriotic cells as well as MMP-2 and -9 activity in primary endometriotic stromal cells in vitro. Reprod. Biol. Endocrinol..

[23] Madanes D (2022). Resveratrol impairs cellular mechanisms associated with the pathogenesis of endometriosis. Reprod. Biomed. Online.

[24] Ricci AG (2013). Natural therapies assessment for the treatment of endometriosis. Hum. Reprod..

[25] Kolahdouz Mohammadi R (2020). The effects of resveratrol treatment on Bcl-2 and bax gene expression in endometriotic compared with non-endometriotic stromal cells. Iran J. Public Health.

[26] Taguchi A (2016). Resveratrol enhances apoptosis in endometriotic stromal cells. Am. J. Reprod. Immunol..

[27] McCormack D, McFadden D (2013). A review of pterostilbene antioxidant activity and disease modification. Oxid. Med. Cell. Longev..

[28] Roupe KA, Remsberg CM, Yáñez JA, Davies NM (2006). Pharmacometrics of stilbenes: Seguing towards the clinic. Curr. Clin. Pharmacol..

[29] Dellinger RW, Gomez Garcia AM, Meyskens FL (2014). Differences in the glucuronidation of resveratrol and pterostilbene: altered enzyme specificity and potential gender differences. Drug Metab. Pharmacokinet..

[30] Zhao G (2022). Polydatin, a glycoside of resveratrol, is better than resveratrol in alleviating non-alcoholic fatty liver disease in mice fed a high-fructose diet. Front. Nutr..

[31] Jiao Y, Wu Y, Du D (2018). Polydatin inhibits cell proliferation, invasion and migration, and induces cell apoptosis in hepatocellular carcinoma. Braz. J. Med. Biol. Res..

[32] Chatterjee K (2018). Resveratrol and pterostilbene exhibit anticancer properties involving the downregulation of HPV oncoprotein E6 in cervical cancer cells. Nutrients.

[33] Shin HJ, Han JM, Choi YS, Jung HJ (2020). Pterostilbene suppresses both cancer cells and cancer stem-like cells in cervical cancer with superior bioavailability to resveratrol. Molecules.

[34] Zhang B, Wang X-Q, Chen H-Y, Liu B-H (2014). Involvement of the Nrf2 pathway in the regulation of pterostilbene-induced apoptosis in HeLa cells via ER stress. J. Pharmacol. Sci..

[35] Schindler CK (2006). Caspase-3 cleavage and nuclear localization of caspase-activated DNase in human temporal lobe epilepsy. J. Cereb. Blood Flow Metab..

[36] Tolomeo M (2005). Pterostilbene and 3′-hydroxypterostilbene are effective apoptosis-inducing agents in MDR and BCR-ABL-expressing leukemia cells. Int. J. Biochem. Cell Biol..

[37] Cregan SP, Dawson VL, Slack RS (2004). Role of AIF in caspase-dependent and caspase-independent cell death. Oncogene.

[38] Arnoult D (2003). Mitochondrial release of AIF and EndoG requires caspase activation downstream of Bax/Bak-mediated permeabilization. EMBO J..

[39] Potter GA (2002). The cancer preventative agent resveratrol is converted to the anticancer agent piceatannol by the cytochrome P450 enzyme CYP1B1. Br. J. Cancer.

[40] Morales P, Haza AI (2012). Selective apoptotic effects of piceatannol and myricetin in human cancer cells. J. Appl. Toxicol..

[41] Westphal D (2014). Apoptotic pore formation is associated with in-plane insertion of Bak or Bax central helices into the mitochondrial outer membrane. PNAS.

[42] Song Z, Han S, Pan X, Gong Y, Wang M (2015). Pterostilbene mediates neuroprotection against oxidative toxicity via oestrogen receptor α signalling pathways. J. Pharm. Pharmacol..

[43] Kobylka P (2022). Resveratrol analogues as selective estrogen signaling pathway modulators: structure-activity relationship. Molecules.

[44] Kawiak A, Kostecka A (2022). Regulation of Bcl-2 family proteins in estrogen receptor-positive breast cancer and their implications in endocrine therapy. Cancers.

[45] Horgan XJ, Tatum H, Brannan E, Paull DH, Rhodes LV (2019). Resveratrol analogues surprisingly effective against triple-negative breast cancer, independent of ERα. Oncol. Rep..

[46] Banu SK, Lee J, Starzinski-Powitz A, Arosh JA (2008). Gene expression profiles and functional characterization of human immortalized endometriotic epithelial and stromal cells. Fertil. Steril..

[47] Logan PC, Ponnampalam AP, Steiner M, Mitchell MD (2013). Effect of cyclic AMP and estrogen/progesterone on the transcription of DNA methyltransferases during the decidualization of human endometrial stromal cells. Mol. Hum. Reprod..

[48] Plaza-Parrochia F (2017). Molecular mechanisms of androstenediol in the regulation of the proliferative process of human endometrial cells. Reprod. Sci..

[49] Bulun SE (2012). Role of estrogen receptor-β in endometriosis. Semin. Reprod. Med..

[50] Jiang Q (2015). Protective effects of polydatin on lipopolysaccharide-induced acute lung injury through TLR4-MyD88-NF-κB pathway. Int. Immunopharmacol..

[51] Lanzilli G (2012). Anti-inflammatory effect of resveratrol and polydatin by in vitro IL-17 modulation. Inflammation.

[52] Bröhan M, Jerkovic V, Collin S (2011). Potentiality of red sorghum for producing stilbenoid-enriched beers with high antioxidant activity. J. Agric. Food Chem..

[53] Shah MA (2022). Uncovering the anticancer potential of polydatin: A mechanistic insight. Molecules.

[54] Su D (2013). Comparision of piceid and resveratrol in antioxidation and antiproliferation activities in vitro. PLoS One.

[55] Hung SW (2021). Pharmaceuticals targeting signaling pathways of endometriosis as potential new medical treatment: A review. Med. Res. Rev..

[56] Swingle KL, Ricciardi AS, Peranteau WH, Mitchell MJ (2023). Delivery technologies for women’s health applications. Nat. Rev. Bioeng..

[57] Olejnik A (2016). Antioxidant effects of gastrointestinal digested purple carrot extract on the human cells of colonic mucosa. Food Chem..

[58] Kowalska K, Dembczyński R, Gołąbek A, Olkowicz M, Olejnik A (2021). ROS modulating effects of lingonberry (*Vaccinium vitis-idaea* L.) polyphenols on obese adipocyte hypertrophy and vascular endothelial dysfunction. Nutrients.

[59] Wendel JRH, Wang X, Smith LJ, Hawkins SM (2020). Three-dimensional biofabrication models of endometriosis and the endometriotic microenvironment. Biomedicines.

